# Prevalence and associated factors of overweight and obesity among reproductive-aged women (15–49 years) in Ratnanagar Municipality, Chitwan district, Nepal

**DOI:** 10.1371/journal.pone.0329850

**Published:** 2025-08-06

**Authors:** Bedana Sapkota, Aashish Rana, Manish Rajbanshi, Richa Aryal, Susmita Thapa, Lokendra Oli, Jiwan Kumar Poudyal

**Affiliations:** 1 Department of Public Health, Shree College of Technology, Purbanchal University, Chitwan, Nepal; 2 Central Department of Public Health, Institute of Medicine, Tribhuvan University, Kathmandu, Nepal; 3 Department of Psychology, Padmakanya Multiple Campus, Tribhuvan University, Kathmandu, Nepal; 4 Department of Public Health and Community Medicine, Chitwan Medical College, Tribhuvan University, Chitwan, Nepal; Nurses' and Midwives Training College, GHANA

## Abstract

**Background:**

Overweight and obesity is a rising public health threat both in developed and developing countries including Nepal. Nepal is undergoing rapid urbanization, accompanied by significant changes in lifestyle, dietary practices, and behavioral patterns. This shift has contributed to a rising prevalence of overweight and obesity. This study aimed to determine the prevalence of overweight and obesity and their associated factors among reproductive-aged women of Ratnanagar Municipality (RM), Chitwan.

**Methodology:**

A cross-sectional study was conducted in selected wards of Ratnanagar Municipality, Chitwan district. A multistage sampling method was followed for the selection of participants. Face-to-face interview was conducted using a structured questionnaire. Data analysis was performed using the Statistical Package for the Social Sciences (SPSS) version 25.0 (IBM). Descriptive results were presented using the frequencies, percentages, mean, and standard deviation. Bivariate analysis was performed using Chi-square and bivariate binary logistic regression. The factors associated with the prevalence of overweight and obesity were determined using a multivariate logistic regression analysis. All the tests were performed at a 95% Confidence Interval and variables with p-values less than 0.05 were considered statistically significant.

**Results:**

A total of 318 individuals participated in this study. More than half of the participants (51.6%) were overweight/obese. This study demonstrated that participant’s socio-demographic and behavioral characteristics such as age (AOR = 3.3, CI: 1.5–4.4), ethnicity (AOR = 2.3, CI:1.2–4.3), higher education (AOR = 2.6 CI:1.8–5.8), family type (AOR = 0.4, CI:0.1–0.8) and moderate physical activity (AOR = 3.0, CI: 1.2–7.4) were statistically significant with the prevalence of overweight and obesity.

**Conclusion:**

This study concludes a significant burden of overweight and obesity (51.6%) among reproductive-aged women, with socio-demographic, lifestyle, and behavioral factors playing a key role. Factors such as age, education, occupation, and physical activity were linked to overweight and obesity. The findings highlight the need for targeted interventions to combat obesity, emphasizing the importance of behavioral changes, public health awareness, and community-based campaigns to promote healthy eating, and reduced sedentary behavior.

## Introduction

Overweight and obesity are rapidly increasing worldwide, and emerging as a public health issue both in developed and developing countries including Nepal [1]. It has emerged as a global pandemic over the past 50 years [2]. According to the World Health Organization (WHO), overweight and obese are the conditions of excessive fat deposits that can impair physical and mental health, and increase the burden of cost [3]. The WHO defines “overweight as a body mass index (BMI) ≥ 25 and obesity as BMI ≥ 30” [3]. Overweight and obesity increases the risk of type-2 diabetes mellitus, cardiovascular diseases (CVDs), gastro-intestinal problems, joint and muscular disorders, respiratory problems, as well as psychological issues [4]. The primary cause of overweight and obesity is an imbalance of energy between calories consumed and calories expended [3]. Additionally, nutritional transition, sedentary lifestyles, urbanization, metabolic and environmental factors are considered to be the main causes of the rise in obesity [5–8]. Previous studies revealed that overweight/obesity was associated with family history of chronic diseases, aging, early marriage, low physical activity, parity, education, place of residence, and wealth status [1,9,10].

Globally, one in eight people is living with obesity, as per the WHO report [3]. Adult obesity has more than doubled since 1990, whereas adolescent obesity has quadrupled worldwide [3]. In 2022, 2.5 billion adults aged 18 years and older were overweight [3]. Evidence indicates that overweight and obesity are more prevalent among women compared to men worldwide [11,12]. It is estimated that the prevalence of overweight and obesity among women is increasing alarmingly in most low- and middle-income countries (LMICs) like Nepal [13]. In many regions of LMICs, the burden of overweight or obesity among women has now exceeded that of underweight, reflecting a shift in the pattern of nutritional health [14]. This is a significant public health concern, as overweight and obesity in reproductive-aged women are associated with serious maternal and child health risks, including gestational diabetes, hypertensive disorders of pregnancy, cesarean deliveries, and complication related to large-for-gestational-age infants [15–17]. Additionally, the nutritional status among reproductive-aged women has far reaching effects on family health and contributes to inter-generational cycles of malnutrition and obesity [18,19].

In Nepal, specifically women of reproductive-aged (15–49 years) are facing rising trend in overweight and obesity [20]. The Nepal Demographic and Health Survey (NDHS) 2022 reported that 35% of women were classified as overweight or obese, showing significant rise from previous years [21]. The Nepal Step wise Approach to Non-communicable Disease Risk Factors Surveillance (STEPS) Survey, 2019 documented an increasing in the trend of obesity among females [22]. Although, there are national-level plans, programs, and approaches such as the Package of Essential Non-Communicable Disease (PEN) Program, and Multisectoral Action Plan for the Prevention and Control of NCDs (2021–2025), the health system of Nepal has been facing challenges in the prevention and control of NCDs due to the diverse geographical, socio-cultural, political and economic factors [23,24]. Despite the escalating rates, there are no specific and targeted interventions aimed at preventing overweight and obesity among reproductive-aged women in Nepal, who represent a particularly vulnerable and influential demographic group.

In Nepal, there are studies and surveys that assess the prevalence and factors affecting overweight and obesity. But, these mostly focus on the general population and there remains a need for further evidence that specifically explores the factors that contribute to the prevalence of overweight and obesity among reproductive-aged women. Furthermore, there is a lack of localized data, particularly at the municipal level, to guide context-specific interventions. Thus, this study aimed to determine the prevalence of overweight and obesity and the factors associated with them among reproductive-aged women in Ratnanagar Municipality (RM) of Chitwan district.

## Materials and methodology

### Study design and setting

A community-based cross-sectional study was carried out among the selected wards of Ratnanagar Municipality of Chitwan district, located in central Terai of Nepal. The total area of Ratnanagar Municipality is 68.67 km^2^ divided into 16 wards and located at 27.59° N latitude and 84.50° E longitude [25]. According to the Nepal Census 2021, there was a total population of 89,905 (male 48.4% and female 51.6%) and more than 30% of the total females belonged to the reproductive-aged group [26].

### Study population

This study was carried out among reproductive-aged women (15–49 years) residing for at least 6 months in this RM. Participants who were not willing to provide consent were excluded from this study. Individuals who were pregnant, lactating, and had communication or hearing problems were excluded. Also, those participants below 18 years of age whose parents were unable to provide consent were excluded from the study.

### Sample size and sampling technique

The sample size was calculated by using the Cochrane proportionate formula (n = Z^2^pq/d^2^) [27]. The prevalence of overweight and obesity among reproductive-aged women (p = 25.4%) was taken from the Nepal STEPS Survey, 2019 [22]. Assuming a 5% allowable error, 95% Confidence Interval (CI), and adding 10% non-response rate, the sample size for this study was 318.

This study followed a multistage sampling method to recruit the participants. Among the total of 16 wards, three wards were randomly selected as the study sites. The three selected wards (Ward 1, Ward 8, and Ward 13) consisted of a total of 4,761 households and these selected wards consisted of n_1_ = 1,428 households, n_8_ = 1,110 households, n_13_ = 2,223 households, respectively. Then, equal proportionate probability sampling was used to determine the sample size for each ward. The sample size for each ward was 106. Similarly, two toles were randomly chosen from the selected wards and 53 households were randomly chosen from each selected toles for the data collection. For the sampling framework, household numbers were identified with the help of the ward office. Female Community Healthcare Volunteers (FCHVs) and local people were mobilized in the identification of respective houses. To minimize non-response bias, follow-up visits were made at different times of the day, and phone contact was used where available. The study objectives were clearly explained to each eligible participant. Support from FCHVs, family members, and caregivers was sought to encourage participation and improve response rates. Nearby households were selected only after at least two unsuccessful visits. If a selected household did not have an eligible participant, the nearest eligible household was approached.

### Tools and measures

A structured questionnaire was adopted from similar studies [1,9]. It was translated into Nepali-language and pre-testing was done among 10% of the sample size (n = 32) in the non-selected ward of RM to check the feasibility and internal consistency of the study tool. This tool was used for the data collection after obtaining a Cronbach’s alpha coefficient of 0.82.

The questionnaire was divided into four sections. It included;

- Section I: It included questions regarding social, economic, and demographical characteristics of the participants such as age, religion, ethnicity, education, occupation, marital status, education and occupation of the husband, family type, and monthly income of the family.
- Section II: This section measured participants’ height (in meters) and weight (in Kg) to determine their Body Mass Index (BMI).
- Section III: It consisted of questions related to physical activities, including frequency of vigorous-intensity activities (per week), frequency of moderate-intensity activities (per week), and frequency of walking at least 10 minutes at a time (per week).
- Section IV: It consisted of questions related to behavioral factors such as eating while watching TV, food consumption as a stress relieving method, alcohol consumption, frequency of breakfast consumption, duration of sleep, use of contraceptive devices, presence of irregular menstrual cycle, use of any kind of medication, and frequency of eating away from home.

### Operational definitions

The BMI was categorized into underweight, normal, overweight and obesity [3].

Underweight: The participants with a BMI less than 18.5 Kg/m^2^ (<18.5 kg/m^2^).

Normal: The participants with a BMI (18.5–24.9 Kg/m^2^).

Overweight: The participants with a BMI (25.0–29.9 Kg/m^2^).

Obesity: The participants with a BMI above 30.0 Kg/m^2^.

Nuclear family: It included family with parents and their children.

Joint family: It included family with parents, children and their grandparents.

Extended family: It included family with parents, children, grandparents, uncle, aunt, cousins and in-laws.

Vigorous physical activity: Activities (like heavy lifting, chopping wood, or digging in the garden) that take hard physical effort and make you breathe much harder than normal [28].

Moderate physical activity: Activities (like carrying light loads, washing, sweeping, cleaning floors) that take moderate physical effort and make you breathe somewhat harder than normal [28].

Illiterate: Those individuals who cannot read and write in any language [29].

### Data collection

The face-to-face interview was carried out to collect the data. Each interview and measurement took approximately 40–45 minutes to complete. The principal investigator was responsible for the data collection procedure and handling of the instruments for data collection. Data collection was conducted between April 1 to July 30, 2024.

Participants’ height was measured using a stadiometer and weight with a digital scale (SECA), validated and recommended by UNICEF [30,31]. Participants’ height was measured following a standardized procedure. They were instructed to remove footwear and head coverings and to stand on a flat surface with their feet together and knees straight. Similarly, weight measurement was conducted with a weighing machine placed on a firm, flat surface. Participants were asked to remove their footwear and wear minimal clothing during the measurement process. Both height and weight were recorded twice, and the average values were calculated for accuracy.

### Data management and analysis

Collected data were systematically entered, filtered, coded, and cross-checked in Epi-Data version 3.1 software. Data analysis was performed using Statistical Package for the Social Sciences (SPSS) version 25.0 (IBM). The participants’ socio-economic and demographic characteristics were described using frequencies, percentages, mean, and standard deviation. Chi-square and Bivariate Binary logistic regression analysis were carried out to measure the association between individual characteristics and overweight/obesity. Multiple regression models were then constructed using different inclusion thresholds (variables with p-values < 0.1, 0.2, and 0.3) and compared with a full model based on theoretical relevance. Then, the final model of variables with p-value < 0.2 was entered into the multivariable logistic regression model to control the effect of confounding variables. The goodness of the test was measured by the Hosmer-Lemeshow Test and the Nagelkerke R-squared value. Variables with p-values less than 0.05 were considered statistically significant. Adjusted odds ratios along with 95% CI were reported to indicate the strength and precision of the associations.

### Ethical statement

This study was reviewed and approved by the Institutional Review Committee, Shree College of Technology Pvt. Ltd., [Reference No: SMTC-IRC-20240318–59]. The letter of support for the data collection was obtained from the Ratnanagar Municipality. Before collecting data, all participants were thoroughly briefed on the study objectives. Both written and verbal consent were obtained from each individual. For participants under the age of 18, written consent was taken from their parents or legal guardians. Participant’s information was kept private and confidential throughout the study period.

## Results

### Socio-demographic characteristics of the study participants

The majority of the participants belonged to the age group 31–40 years (36.8%), Hindu religion (87.4%), Janajati ethnicity (57.9%), completed secondary-level education (25.8%), married (82.1%), and belonged to the nuclear families (77.0%). The majority of the participants (41.5%) were housemakers. Most of the participant’s families (38.4%) had a monthly income ranging between NRs 25,000–50,000 (Table 1).

**Table 1:** Socio-demographic characteristics of the participants.

Characteristics	Number (n)	Percentage (%)
**Age (in years)**		
Less than 20	36	11.3
20–30	93	29.3
31–40	118	37.1
41–49	71	22.3
**Religion**		
Hindu	278	87.4
Christian	20	6.3
Buddhist	12	3.8
Islam	8	2.5
**Ethnicity**		
Brahmin/Chhetri	93	29.2
Janajati	184	57.9
Dalit	33	10.4
Muslim	8	2.5
**Marital status**		
Married	261	82.1
Unmarried	57	17.9
**Education level**		
Illiterate	57	17.9
Can read and write	72	22.6
Primary level	67	21.1
Secondary level	82	25.8
Bachelor and above	40	12.6
**Occupation**		
Homemaker	132	41.5
Agriculture	67	21.1
Business	58	18.2
Student	49	15.4
Service (Government employment/INGO/NGO)	12	3.8
**Education level of the husband of married women, (n = 261)**		
Illiterate	39	14.9
Can read and write	65	24.9
Primary level	71	27.2
Secondary level	48	18.4
Bachelor and above	38	14.6
**Occupation of the husband of married women, (n = 261)**		
Foreign employment	76	29.1
Labour	56	21.5
Business	55	21.1
Agriculture	53	20.3
Service	12	4.6
Others	9	3.4
**Family type, (n = 318)**		
Nuclear	245	77.1
Extended	24	7.5
Joint	49	15.4
**The monthly income of the family** (1USD = NRs.138.57)		
Less than NRs 25,000	93	29.2
NRs 25,000–50,000	122	38.4
NRs 50,000–1,00,000	90	28.3
More than NRs 1,00,000	13	4.1

### BMI, physical activity, and behavioral-related activities of the participants

The majority of participants (41.8%) had a normal BMI, followed by those who were overweight (30.2%) and obese (21.4%). Most participants (81.1%) did not engage in vigorous physical activity, and less than half (46.5%) reported walking at least 10 minutes daily. Over one-third (36.4%) avoided eating while watching TV, and 8.8% consumed food as a method of stress relief. Nearly two-thirds (64.0%) of the participants either had irregular breakfast habits or never consumed breakfast. Most participants (82.1%) reported sleeping at least eight hours per day. The majority (88.5%) did not use any contraceptive method. Moreover, more than half (58.5%) reported eating away from home either twice a week or three to four times a week (Table 2).

**Table 2:** BMI, physical activity and behavioral-related activities of the participants.

BMI	Number (n)	Percentage (%)
Underweight	21	6.6
Normal	133	41.8
Overweight	96	30.2
Obesity	68	21.4
**Vigorous physical activity (last 7 days)**		
Yes	56	18.9
No	241	81.1
**Moderate physical activity (last 7 days)**		
Yes	267	89.9
No	30	10.1
**Walk at least 10 minutes per day (last 7 days)**		
Yes	138	46.5
No	159	53.5
**Eating while watching TV**		
Daily	68	22.9
Twice in a week	65	21.9
3–4 times a week	56	18.8
Never	108	36.4
**Food as a stress relieving method**		
Yes	26	8.8
No	271	91.2
**Consume alcohol**		
Yes	9	3.0
No	288	97.0
**Consume breakfast**		
Never	123	41.4
Irregular	67	22.6
Daily	107	36.0
**Sleep duration (per day)**		
≥ 8 hours	244	82.1
< 8 hours	53	17.9
**Use of contraceptive devices**		
Yes	34	11.5
No	263	88.5
**Irregular menstrual cycle**		
Yes	49	16.5
No	248	83.5
**Frequency of eating away from home (in a week)**		
Once	58	19.5
2 times	110	37.0
3–4 times	64	21.5
More than 4 times	30	10.2
Never	35	11.8

### Association between socio-demographic characteristics and overweight/obesity

This study showed higher odds of overweight/obesity among participants aged 40 and above, i.e., (AOR = 3.3, 95% CI; 1.5–4.4). Participants who belonged to the Janajati ethnicity were 2.3 times more likely to be overweight/obese (AOR = 2.3 CI: 1.2–4.3). The participants who had a higher level of education and belonged to the nuclear family showed higher odds of being overweight/obese (Table 3).

**Table 3:** Association between socio-demographic characteristics and overweight/obesity.

Characteristics	Overweight/Obesity		COR, 95% CI	p-value	Multivariate logistic regression	p-value
	Yes	No			AOR, 95% CI	
	n (%)	n (%)				
**Age (in years)**						
40 and above	55 (77.5)	16 (22.5)	3.6 (1.2-4.3)		3.3 (1.5-4.4)	
Less than 40	109 (48.2)	117 (51.8)	Ref	0.000	Ref	0.003
**Religion**						
Hindu	155 (57.4)	115 (42.6)	2.6 (1.2-3.4)		2.3 (1.2-3.1)	
Non-Hindu	9 (33.3)	18 (66.7)	Ref	0.020	Ref	0.014
**Ethnicity**						
Janajati	103 (50.5)	101 (49.5)	1.8 (1.1-3.1)		2.3 (1.2-4.3)	
Others	61 (65.6)	32 (34.4)	Ref	0.016	Ref	0.009
**Educational level**						
Secondary or above	75 (57.3)	56 (42.7)	1.9 (0.9-3.7)		2.6 (1.8-5.8)	
Primary or below	53 (48.2)	57 (51.8)	1.3 (0.7-2.5)		1.5 (0.7-3.2)	
Illiterate	36 (64.3)	20 (35.7)	Ref	0.120	Ref	0.049
**Occupation of women**						
Housemaker	46 (65.7)	24 (34.3)	1.7 (1.2-1.9)		1.5 (1.2-1.8)	
Others	118 (52.0)	109 (48.0)	Ref	0.045	Ref	0.085
**Marital status**						
Married	159 (54.6)	132 (45.4)	Ref	0.196	Ref	0.525
Others	5 (83.3)	1 (16.7)	4.1(0.4-35.9)		2.0 (0.2-19.7)	
**Educational level of the husband**						
Secondary or above	65 (50.0)	65 (50.0)	1.5 (0.7-3.2)			
Primary level	76 (58.9)	53 (41.1)	1.0 (0.5-2.2)			
Illiterate	23(60.5)	15 (39.5)	Ref	0.277		
**Occupation of husband**						
Agriculture	26 (53.1)	23 (46.9)	Ref	0.769		
Labour	31 (55.4)	25 (44.6)	0.9 (0.4-1.9)			
Business	33 (60.0)	22 (40.0)	0.7 (0.3-1.6)			
Foreign employment	42 (58.3)	30 (41.7)	0.8 (0.3-1.6)			
Others	32 (49.2)	33 (50.8)	1.1 (0.5-2.4)			
**Family type**						
Nuclear	146 (57.9)	106 (42.1)	Ref	0.028	Ref	0.017
Joint/extended	18 (40.0)	27 (60.0)	0.4 (0.2-0.9)		0.4 (0.1-0.8)	
**Monthly income of family (in NRs), 1 USD = NRs 135.73**						
50,000 or above (≥USD 369.90)	54 (55.1)	44 (44.9)	1.1 (0.6-1.6)			
Less than 50,000 (<USD 369.90)	110 (55.3)	89 (44.7)	Ref	0.977		

Ref: reference.

COR: Crude Odds Ratio, AOR: Adjusted Odds Ratio.

### Association of physical activity and behavioral factors with overweight/obesity

The study revealed that participants who did not engage in moderate physical activity had significantly higher odds of being overweight or obese (AOR = 3.0, 95% CI: 1.2–7.4). However, the findings indicated that no other behavioral factors showed a significant association with overweight or obesity (Table 4).

**Table 4:** Association of physical activity, behavioral factors with overweight/obesity.

Characteristics	Overweight/Obesity		COR, 95% CI	p-value	Multivariate logistic regression	p-value
	Yes	No			AOR, 95% CI	
	n (%)	n (%)				
**Vigorous physical activity**						
Yes	33 (58.9)	23 (41.1)	0.8 (0.4-1.4)			
No	131 (54.4)	110 (45.6)	Ref	0.536		
**Moderate physical activity**						
Yes	143 (53.6)	124 (46.4)	Ref	0.091	Ref	0.017
No	21 (70.0)	9 (30.0)	2.0 (0.8-4.5)		3.0 (1.2-7.4)	
**Walk at least 10 minutes (per day)**						
Yes	78 (56.5)	60 (43.5)	1.1 (0.6-1.7)			
No	86 (54.1)	73 (45.9)	Ref	0.726		
**Eating while watching TV**						
Daily	32 (47.1)	36 (52.9)	1.7 (0.9-3.1)		1.8 (0.9-3.5)	
Twice in a week	32 (49.2)	33 (50.8)	1.5 (0.8-2.8)		0.7 (0.3-1.6)	
3–4 times a week	35 (62.5)	21 (37.5)	0.9 (0.4-1.7)		1.2 (0.6-2.5)	
Never	65 (60.2)	43 (39.8)	Ref	0.171	Ref	0.188
**Food as a stress relieving method**						
Yes	15 (57.7)	11 (42.3)	1.1 (0.4-2.5)			
No	149 (55.0)	122 (45.0)	Ref	0.839		
**Consume alcohol**						
Yes	4 (44.4)	5 (55.6)	0.6 (0.1-2.4)			
No	160 (55.6)	128 (44.4)	Ref	0.736		
**Consume breakfast**						
Never	65 (52.8)	58 (47.2)	1.2 (0.7-2.0)			
Irregular	37 (55.2)	30 (44.8)	1.1 (0.6-2.0)			
Daily	62 (57.9)	45 (42.1)	Ref	0.740		
**Sleep duration**						
< 8 hours	134 (54.9)	110 (45.1)	0.9 (0.5-1.7)			
≥ 8 hours	30 (56.6)	23 (43.4)	Ref	0.880		
**Use of contraceptives**						
Yes	16 (47.1)	18 (52.9)	0.6 (0.3-1.4)			
No	148 (56.3)	115 (43.7)	Ref	0.361		
**Irregular menstrual cycle**						
Yes	26 (53.1)	23 (46.9)	0.9 (0.4-1.6)			
No	138 (55.6)	110 (44.4)	Ref	0.755		
**Eating away from home (in a week)**						
Once	31 (53.4)	27 (46.6)	0.7 (0.3-1.7)			
2 times	67 (60.9)	43 (39.1)	0.5 (0.2-1.1)			
3–4 times	35 (54.7)	29 (45.3)	0.6 (0.3-1.5)			
More than 4 times a week	15 (50.0)	15 (50.0)	0.8 (0.3-2.2)			
Never	16 (45.7)	19 (54.3)	Ref	0.540		

Ref: reference.

COR: Crude Odds Ratio, AOR: Adjusted Odds Ratio.

## Discussion

Overweight and obesity have been emerging as a major public health problem in Nepal, affecting all age groups [32]. This study revealed that more than half of reproductive-aged women (51.1%) were overweight or obese. A previous study among reproductive-aged women in Eastern Nepal found that the prevalence of overweight/obesity was nearly 42% [33]. Another study in Dharan Sub-Metropolitan City, Nepal, found that nearly 51% of reproductive-aged women were overweight/obese [1]. This finding is similar to several studies conducted in other Asian countries like India, Bangladesh, and Pakistan [9,34,35]. The higher prevalence of overweight/obesity among women is related to various factors such as nutritional transition (shift from traditional diets and lifestyles to Western diets), cultural and behavioral shifts, sedentary behavior, and socio-economic progress [10,12,36,37]. Globalization has influenced the dietary and lifestyle pattern among people by food promotion, cultural exchange, increased availability of ultra-processed foods, improved socio-economic status, etc [8,38]. This phenomenon has significantly contributed to the increasing prevalence of obesity.

This study demonstrated that women aged 40 and above were at 3.3 times higher risk of being overweight or obese compared to those under 40 years. The finding is in the line with previous studies carried out in Cambodia, Bangladesh, and Nepal [34,39,40]. The higher risk among older women is because, with the increase in age, women are more vulnerable to unhealthy diet consumption and lack of physical activity [39,41]. Another potential explanation is that as individuals grow older, their body composition changes leading to an increase in fat mass and a reduction in fat-free mass [42,43].

This study showed that ethnicity was significantly associated with overweight/obesity and the finding is coherent with the study conducted in India [9]. This study showed that Janajati were 2.3 times more likely to be overweight/obese. Similarly, a previous study found that ethnicity was associated with lifestyle and behavioral factors that contribute to increased risk of overweight and obesity [44]. The increased risk of overweight or obesity among some ethnic groups can be linked to cultural differences, dietary habits, and lifestyle practices. Certain communities may traditionally consume diets rich in carbohydrates or fats, while higher alcohol and tobacco consumption in some Indigenous groups may further contribute to weight gain and associated health risks [45,46].

Women with a higher education level were 2.6 times more likely to be overweight/obese than those with lower education. Similar findings have been reported in the previous studies conducted in Nepal, India, and Bangladesh [9,40,47]. This trend can be attributed to women with higher education having better job opportunities and working environments, often leading to occupations that involve less physical activity [12,47]. Additionally, educated women are often wealthier, leading to a more sedentary lifestyle and the ability to afford highly caloric food items [9].

In the current study, women’s occupational status was found to be significantly associated with overweight/obesity. The findings explained that housemakers were 1.7 times more likely to be overweight or obese compared to women engaged in other occupations. Similar findings were found in the previous studies in Iran and Bangladesh [48,49]. Housemakers are often concerned with sedentary lifestyle and with limited physical activity as their daily routines may involve prolonged periods of sitting or minimal movement [48,50]. Additionally, housemakers may have dietary patterns that contribute to obesity, such as consuming high-calorie foods and irregular meal timings. Furthermore, housemakers are more vulnerable to mental health issues such as anxiety, depression, and emotional stress and these conditions can significantly contribute to unhealthy eating habits, stress-related eating, and subsequent weight gain [51,52].

Furthermore, the current study revealed that women belonging to the nuclear families had higher odds of being overweight or obese compared to those from other family structures. Nuclear families may experience economic constraints that limit access to healthy foods and recreational activities, contributing to weight gain [53]. Due to their smaller household size and time constraints, nuclear families might prioritize quick and easy meal options, which are often calorie-dense and nutritionally imbalanced [53]. These factors contribute to an increased risk of overweight and obesity.

This study found no significant association between household monthly income and overweight/obesity. However, women with a household monthly income of NRs 50,000 or more had 1.1 times higher odds of being overweight or obese compared to those earning less than NRs 50,000. In contrast, previous studies have reported a positive association between income and overweight/obesity [34,54].

Individuals who did not participate in moderate physical activity were 3 times more likely to be overweight or obese compared to their counterparts. This finding aligns with evidence generated from previous studies [55,56]. Physical exercise not only burns calories but also enhances lipid oxidation, which may promote carbohydrate utilization for energy while maintaining a balanced energy intake, ultimately supporting a normal BMI [55,57].

This study concluded that those who eat while watching TV were more likely to be overweight/obese. This behavior may contribute to overeating, as watching TV can distract individuals from recognizing feelings of fullness, leading to a high intake of calories. Additionally, watching TV is often associated with exposure to food advertisements, which can influence cravings and encourage the consumption of unhealthy and calorie-dense snacks [58].

The study also depicted that behavioral factors like using food as a stress-relieving method increase the risk of being overweight/obese. This result is supported by the previous studies and shows that stress influences human eating behavior, and can lead to both hyperphagia and hypophagia [51,59]. Stress-induced eating is one of the contributing factors to overweight/obesity [59].

In this study, women who slept 8 hours or more a day had higher odds of being overweight/obese than those who slept for less than 8 hours. This finding is consistent with findings from a previous study and explains that excessive sleep was linked to a slower metabolism and weight gain [60]. Sleeping for prolonged periods reduces overall physical activity and fewer calories burned, which can contribute to weight gain [61].

Furthermore, this study showed that those who consumed breakfast daily were less likely to be overweight/obese. It might be that individuals who regularly eat breakfast are more likely to maintain a structured daily routine and healthier eating habits overall. Skipping breakfast often leads to increased hunger, which may result in high food consumption or overeating. Previous studies showed that daily breakfast consumption was associated with a lower risk of overweight and obesity [62,63].

### Strengths and limitations

This study has several strengths and limitations. Firstly, a validated tool was used for data collection, ensuring reliability. It comprehensively assessed various factors influencing overweight and obesity, including social, economic, lifestyle, behavioral, dietary, and environmental determinants. Furthermore, the study utilized the WHO classification of BMI to assess nutritional status, aligning with national surveys such as NDHS. This consistency enhances the generalizability of the findings in comparison to previous studies.

Besides several strengths, it has some limitations. This study did not consider non-modifiable risk factors like genetics, family history, past medical history, etc. as determinants of overweight and obesity, that might influence the study findings.

## Conclusion

This study highlights the significant burden of overweight and obesity (51.6%) among reproductive-aged women. Several socio-demographic, lifestyle, and behavioral factors were found to be significantly associated with overweight and obesity. Age, ethnicity, education, occupation, family structure, physical activity, and sleep duration were all identified as contributing factors. The study underscores the importance of targeted interventions to address the growing obesity epidemic, particularly among women who are more vulnerable due to lifestyle changes, cultural influences, and occupational roles. The association between a sedentary lifestyle and weight gain suggests the need for increased awareness and behavioral modifications to promote healthier living. This study highlights the importance of public health awareness programs and the implementation of community-based awareness campaigns focusing on healthy eating habits and the risks associated with excessive calorie intake and a sedentary lifestyle. Moreover, interventions should include community-based programs such as health awareness workshops, nutrition counseling, and regular physical activity promotion through local health facilities. Policy-level changes, including regulating the marketing of unhealthy foods and creating walkable spaces, would also support long-term behavioral change and improved public health outcomes.

## Supporting information

S1 Data Data set for analysis. (XLSX)
